# On the formation of fold-type oscillation marks in the continuous casting of steel

**DOI:** 10.1098/rsos.170062

**Published:** 2017-06-07

**Authors:** M. Vynnycky, S. Saleem, K. M. Devine, B. J. Florio, S. L. Mitchell, S. B. G. O’Brien

**Affiliations:** 1 Department of Materials Science and Engineering, KTH Royal Institute of Technology, Brinellvägen 23, 100 44 Stockholm, Sweden; 2 Mathematics Applications Consortium for Science and Industry (MACSI), Department of Mathematics and Statistics, University of Limerick, Limerick, Ireland

**Keywords:** continuous casting, oscillation marks, asymptotic analysis

## Abstract

Asymptotic methods are employed to revisit an earlier model for oscillation-mark formation in the continuous casting of steel. A systematic non-dimensionalization of the governing equations, which was not carried out previously, leads to a model with 12 dimensionless parameters. Analysis is provided in the same parameter regime as for the earlier model, and surprisingly simple analytical solutions are found for the oscillation-mark profiles; these are found to agree reasonably well with the numerical solution in the earlier model and very well with fold-type oscillation marks that have been obtained in more recent experimental work. The benefits of this approach, when compared with time-consuming numerical simulations, are discussed in the context of auxiliary models for macrosegregation and thermomechanical stresses and strains.

## 1. Introduction

**Figure 1.**
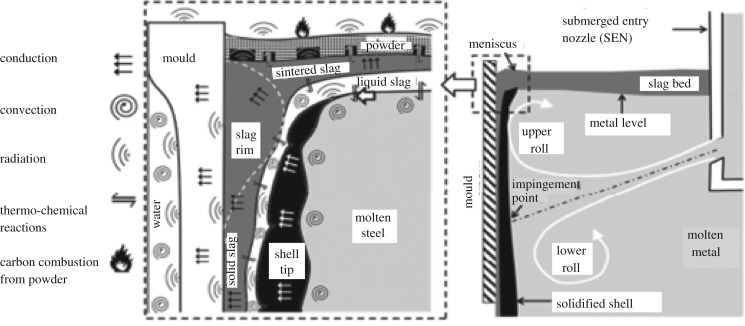
Schematic diagram of the start of casting at the meniscus region.

Mould oscillation has been implemented for a long time in the continuous casting of steel in order to avoid sticking of the solid shell to the mould walls if there is insufficient lubrication. However, oscillation leads inevitably to the formation of regularly spaced indentations along the slab width, known as oscillation marks. A schematic of the situation is shown in figure 1.

**Figure 2.**
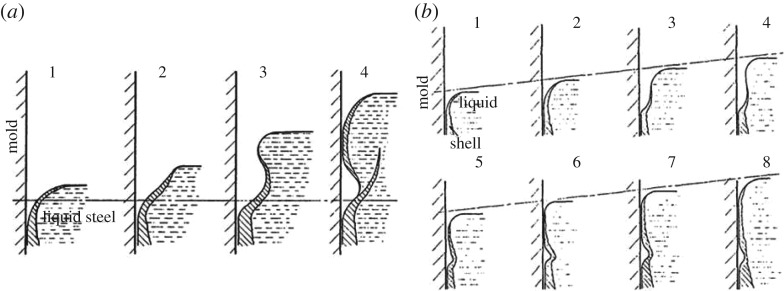
Diagrams showing proposed mechanisms for the formation of oscillation marks, adapted from Tomono [1]: (*a*) overflow, where liquid metal overflows the solidified meniscus; and (*b*) folding, where the shell appears to bend back during the part of the cycle when the velocity of the mould in the casting direction is greater than the casting speed.

The nature of these marks has long been a subject of modelling in the continuous casting literature [2]; a recent and comprehensive literature review is given in [3] and is, therefore, not repeated here. However, it is worth highlighting that, since the early work of Tomono [1], oscillation marks have, in general, been classified as either overflow-type or fold-type; the difference is demonstrated in figure 2. In both cases, the shell solidifies along the curved meniscus profile to form a pointed tip with a hook shape. The critical difference lies in the thickness of the shell. In figure 2*a*, the shell is strong enough to avoid deformation, causing the steel meniscus to overflow the tip, as shown. Otherwise, if the shell is too thin, its tip bends back under the rim pressure, as shown in figure 2*b*. It should also be pointed out that these figures are based on experiments using an organic solvent that is teemed into a mould region; consequently, in both cases, the level of the meniscus rises according to the numerical sequence given in each sub-figure. Thus, although the configuration is not exactly identical to that in continuous casting, the observations have nevertheless been used to posit mechanisms for oscillation-mark formation in this process. More recently, attempts at modelling this problem have tended towards resource-heavy computational fluid dynamics [3–7], which appear to capture the salient details of the phenomena, although at great computational cost. There are a number of difficulties with this approach. First, since one computational run with a given combination of operating parameters—casting speed, mould oscillation frequency, the amplitude of mould oscillations, also referred to as the stroke, and the flux viscosity and so on—is so time-consuming, it is clear that parameter studies are even more so; hence, modelling becomes an unwieldy tool for understanding oscillation-mark formation. Secondly, in view of the need to understand important phenomena that are known to occur in the vicinity of oscillation marks, such as macrosegregation and thermal cracking, it will clearly be difficult to extend such numerical models to include these effects.

In this context, we revisit an earlier model for oscillation-mark formation by Hill *et al.* [8], who had built on yet earlier work by Bland [9], King *et al.* [10] and Fowkes *et al*. [11,12] by using lubrication theory coupled to heat conduction, so as to predict the solid and liquid slag thickness and the oscillation-mark shape. However, while we believe that the model of Hill *et al.* [8] would be a good starting point for the modelling of segregation and thermal stresses, it is also apparent that it needs to be reanalysed first, since a number of unquantified assumptions were made in the course of its derivation, and neither was it non-dimensionalized in any systematic way. In summary, then, the purpose of this article is to give a systematic analysis and understanding of the earlier model by Hill *et al.* [8], with a view to providing an experimentally validated theoretical model that is not so time-consuming to use.

The layout of this paper is as follows. In §2, we summarize recently obtained experimental observations on oscillation-mark formation that are of relevance for the theoretical part of this study. In §3, we recap the oscillation-mark model formulated in [8], and in §4 we non-dimensionalize it. We arrive at a model with 12 dimensionless parameters; it turns out that, based on the physical parameters of the problem, some of the earlier assumptions regarding which terms can be neglected are not formally valid. In §5, we give what we believe should be the correct asymptotically reduced model. Deferring the numerical solution of this asymptotically reduced model to future work, in §6, we instead relax the values of some of the dimensionless parameters to obtain a more analytically tractable problem that nevertheless retains the qualitative features of the reduced model in §5. The results are given in §7, and give particularly good agreement with recent experimental observations, as well as with the results in [8]. Conclusions are drawn in §8.

## 2. Experiment

Industrial plant trials were performed using a slab continuous caster with cross section 290×1500 mm and a mould oscillating in sinusoidal mode with fixed stroke. The casting conditions, with respect to casting speed, *V*_cast_, mould oscillation frequency, *ω*/2*π*, where *ω* is the angular frequency, and stroke, *a*, are given or can be calculated from table 1; in particular, *a* is related to the maximum oscillation velocity, *V*_0_, by *a*=2*πV*_0_/*ω*. Further details of the steel alloy used, which are not necessary for the model to be presented here, are given by Saleem [13].

**Table 1.** Model parameters from Hill *et al.* [8].

symbol	value	unit
*C*^(l)^_f_	1260	J kg^−1^ K^−1^
*C*^(s)^_f_	1260	J kg^−1^ K^−1^
*C*^(s)^_s_	670	J kg^−1^ K^−1^
*k*^(l)^_f_	1.5	W m^−1^ K^−1^
*k*^(s)^_f_	2.25	W m^−1^ K^−1^
*k*^(s)^_s_	30^*a*^	W m^−1^ K^−1^
*g*	9.81	m s^−2^
l	0.298,0.7^*b*^	m
m	10^4^,8.5×10^3^^*b*^	W m^−2^ K^−1^
*R*	2×10^−4^	m^2^ K W^−1^
*T*_f,s_	1373	K
*T*_m,s_	1773	K
*T*_w_	300	K
*V*_cast_	0.0045, 0.013^*b*^	m s^−1^
*V*_0_	0.0126,0.029^*b*^	m s^−1^
Δ*H*_s_	272 000	J kg^−1^
*μ*_f_	0.5, 0.07^*b*^	Pa s
*ρ*^(l)^_f_	2930	kg m^−3^
*ρ*^(s)^_f_	2930	kg m^−3^
*ρ*^(s)^_s_	7800	kg m^−3^
*ω*	4*π*/3,19*π*/6^*b*^	rad s^−1^

^*a*^Mills *et al.*[17].^*b*^Alternative values from Saleem [13].

**Figure 3.**
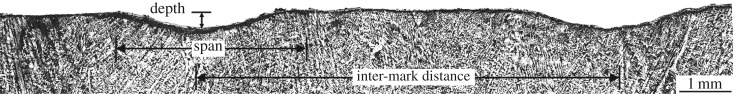
Two adjacent oscillation marks and typically observed microstructure underneath them.

Samples with length 250 mm and cross section 10×10 mm were cut mechanically from the narrow face of the strand in order to avoid the possible effect of soft reduction and distortion in the as-cast outer contour of the chill surface. The oil and oxidized surface layer on the etched and unetched surfaces of samples were first removed with a wire metal brush to expose the existing cast surface. The specimens were ground and polished by standard surface finish for micro-examinations. The specimens then were micro-etched with a solution of 4–9 g picric acid in 100 ml H_2_O, and were then cleaned in an ultrasonic cleaner by placing them in a solution of (3 ml) HCl, (50 ml) water and (4 ml) 2 Butyne-1 diol, followed by gentle polishing for 20 s. The etchant used for the material makes it possible to see the primary microstructure; an example of this is given in figure 3, which shows two adjacent oscillation marks with dendrites beneath the surface. Although the figure shows only two marks, all of the marks obtained for this alloy and under these casting conditions were found to have this general form, which, in the context of the relevant literature [2,7,14,15], is categorized as a fold-type mark, as distinct from an overflow-type mark. Moreover, photographing continuously along the entire length and exporting the images to-scale to drafting software AutoCAD enabled the analysis of the geometrical features of the outer contour of the surface; we will present some results of this later in §7.2, when we make a comparison with theory.

**Figure 4.**
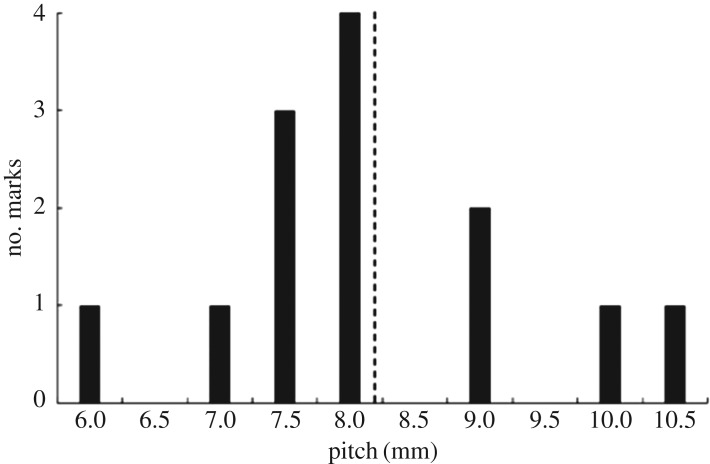
Experimental results showing the number of marks associated with a given pitch. The vertical dashed line shows the average value.

For completeness, we show in figure 4 the entire dataset of measured oscillation-mark spacings—commonly known as the pitch—that were obtained. From this, we see a total of 13 marks were analysed and that, more often than not, the pitch obtained was close to the average value; this may in itself not be noteworthy; other than that this is also close to the theoretical value, as seen later in §7. In particular, the marks shown in figure 3 correspond to two of the marks at 8 mm in figure 4.

## 3. Mathematical model

**Figure 5.**
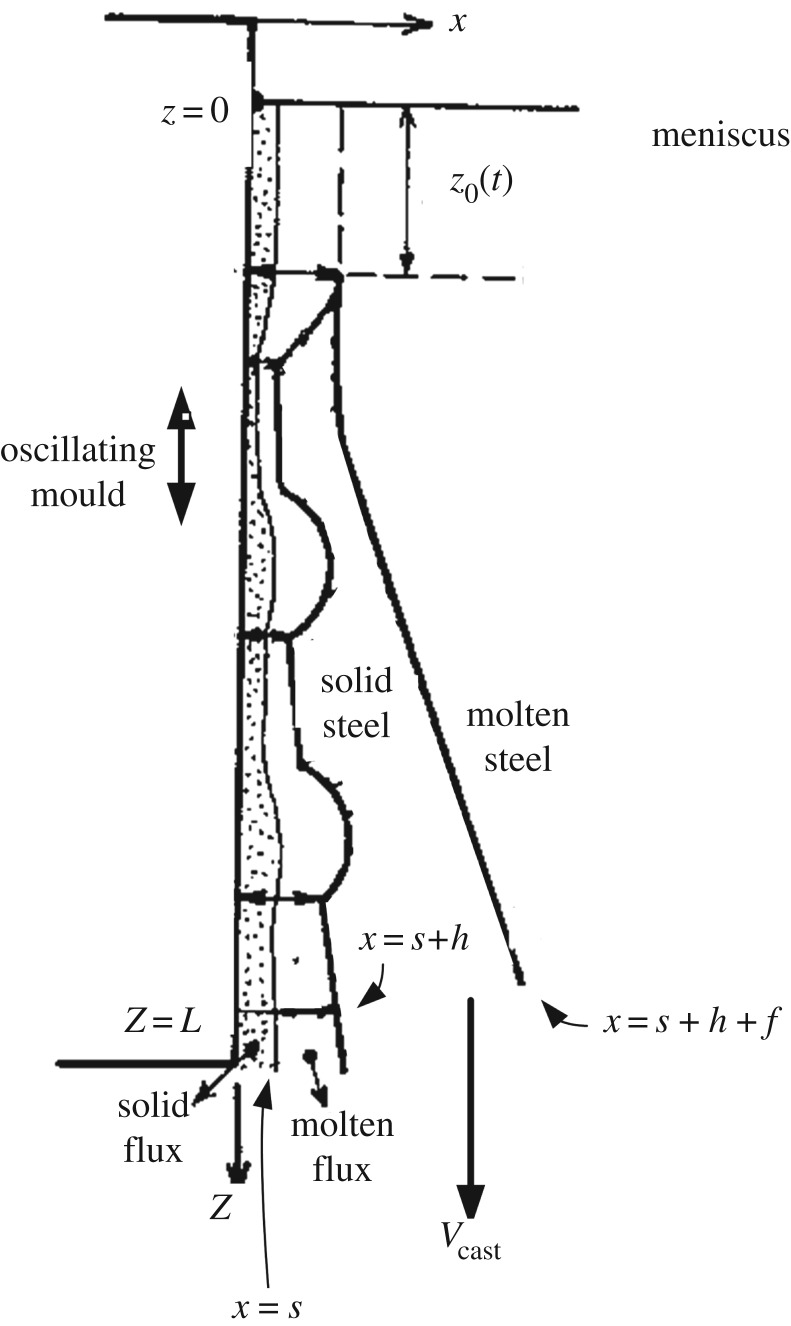
Schematic diagram showing the process of oscillation-mark formation.

Now, we turn to the derivation of a mathematical model to describe the formation of the oscillation marks which were documented in §2. To this end, we consider the time-dependent two-dimensional formulation of Hill *et al.* [8], a schematic for which is shown in figure 5. For *x*<0, there is a mould, cooled by water at temperature *T*_w_ that flows through horizontally oriented pipes, that oscillates in the vertical *z*-plane with speed *V* (*t*), where *t* is the time. The mould cools a layer of molten flux powder that is fed vertically down adjacent to it; some of the flux solidifies, occupying the region 0<*x*<s(*z*,*t*), whereas the rest remains molten and occupies s(*z*,*t*)<*x*<s(*z*,*t*)+*h*(*z*,*t*); the interface at *x*=s is taken to be at the melting temperature of the flux, *T*_m,f_. Further out, the region *x*>s(*z*,*t*)+*h*(*z*,*t*)+f(*z*,*t*) is occupied by molten steel which starts to solidify at a distance *z*_0_(*t*) below the meniscus, and at a distance s_0_(*t*)+*h*_0_(*t*) from the oscillating mould; thus, this point can move with time, and note also that the molten flux/molten steel interface is assumed to be vertical for 0<*z*<*z*_0_(*t*). Moreover, we remark here that that knowledge of *z*_0_(*t*) turns out to be irrelevant for the oscillation-mark formation mechanism according to the present model, as it will be eliminated by an asymptotic argument following equation (6.37). The interface at *x*=*s*+*h*+f separates the solid and molten steel and is assumed to be at the melting temperature of steel, *T*_m,s_; moreover, the solid steel, which occupies the region *s*+*h*<s<*s*+*h*+f is withdrawn vertically down in the *z*-direction with a uniform speed, *V*_cast_. Note also that the solidified steel shell can also be subject to thermal contraction, although we neglect this effect here, as it seemed to make little contribution to the results for the computed oscillation-mark profile in [8]; had the contribution been more significant, the profile in question, in their figure 5, would have more closely resembled the schematic in their figure 3 (and our figure 5).

In what follows, we give the governing equations in §3.1, and the boundary and initial conditions in §3.2.

### 3.1. Governing equations

The problem is then divided into a lower zone, where *z*>*z*_0_(*t*), and an upper zone where *z*<*z*_0_(*t*). In §i below, we give the governing equations for the lower zone; after that, in §ii, we give the equations for the upper zone.

#### 3.1.1. Lower zone

For 0<*x*<s(*z*,*t*), heat transfer is governed by

3.1 $$\rho_{f}^{\left(s\right)}c_{f}^{\left(s\right)}\left(\frac{\partial T}{\partial t}+V\frac{\partial T}{\partial z}\right)=k_{f}^{\left(s\right)}\frac{\partial^{2}T}{\partial x^{2}},$$

where *T* is the temperature and $c_{f}^{\left(s\right)},k_{f}^{\left(s\right)}$ and $\rho_{f}^{\left(s\right)}$ are, respectively, the specific heat capacity, the thermal conductivity and the density of the solid flux; typically, the mould oscillates according to

3.2 $$V(t)=V_{0}\cos\omega t$$

and the solid flux is thus assumed to oscillate with it. In writing (3.1), we have already assumed that the geometry we consider is slender, and we have, therefore, neglected heat conduction in the *z*-direction; we will do likewise for the remaining regions.

For s(*z*,*t*)<*x*<s(*z*,*t*)+*h*(*z*,*t*), conservation of mass, momentum in the *z*-direction and heat are expressed, respectively, by

3.3 $$\begin{array}{rl} & \frac{\partial u_{x}}{\partial x}+\frac{\partial u_{z}}{\partial z}=0, \end{array}$$

3.4 $$\begin{array}{rl} & 0=-\frac{\partial p}{\partial z}+\frac{\partial }{\partial x}\left(\mu_{f}\frac{\partial u_{z}}{\partial x}\right)+\rho_{f}^{\left(l\right)}g \end{array}$$

3.5 $$\begin{array}{rl} \text{and}\; & \rho_{f}^{\left(l\right)}c_{f}^{\left(l\right)}\left(\frac{\partial T}{\partial t}+u_{x}\frac{\partial T}{\partial x}+u_{z}\frac{\partial T}{\partial z}\right)=k_{f}^{\left(l\right)}\frac{\partial^{2}T}{\partial x^{2}}, \end{array}$$

where $c_{f}^{\left(l\right)},k_{f}^{\left(l\right)}$, *ρ*^(l)^_f_ and *μ*_f_ are, respectively, the specific heat capacity, the thermal conductivity, the density and viscosity of the molten flux, *u*_*x*_ and *u*_*z*_ are the molten flux velocities in the *x*- and *z*-directions, respectively, *p* is the pressure and *g* is the gravitational acceleration. For (3.5), we have employed the usual lubrication approximation, whereby the inertia terms are neglected, which leads to ∂*p*/∂*z* being a function of *z* and *t*; we analyse the validity of this approximation in §4, once dimensionless parameters have been introduced.

For s(*z*,*t*)+*h*(*z*,*t*) <*x*<s(*z*,*t*)+*h*(*z*,*t*)+f(*z*,*t*), the heat equation is written as

3.6 $$\rho_{s}^{\left(s\right)}c_{s}^{\left(s\right)}\left(\frac{\partial T}{\partial t}+V_{\mathrm{cast}}\frac{\partial T}{\partial z}\right)=k_{s}^{\left(s\right)}\frac{\partial^{2}T}{\partial x^{2}},$$

where $c_{s}^{\left(s\right)},k_{s}^{\left(s\right)}$ and *ρ*^(s)^_s_ are, respectively, the specific heat capacity, the thermal conductivity and the density of the solid steel.

Lastly, we point out that we do not solve any equation for the molten steel region, but will simply include its effect in terms of a heat flux prescribed at *y*=s(*z*,*t*)+*h*(*z*,*t*)+f(*z*,*t*). Moreover, later scaling arguments indicate that whereas its prescription will affect the location of the molten steel/solid steel interface, it will not affect the location of the oscillation marks themselves.

#### 3.1.2. Upper zone

For the upper zone, only the *z*-direction momentum equation is considered [8,9,11,12], so that we have just

3.7 $$0=-\frac{\partial p}{\partial z}+\frac{\partial }{\partial x}\left(\mu_{f}\frac{\partial u_{z}}{\partial x}\right)+\rho_{f}^{\left(l\right)}g.$$

### 3.2. Boundary and initial conditions

A heat balance at *x*=0 gives

3.8 $$k_{f}^{\left(s\right)}\frac{\partial T}{\partial x}=\frac{m}{1+mR_{\mathrm{mf}}}(T-T_{w}),$$

where *T*_w_ is the pipe water temperature, *R*_mf_ is the interface thermal contact resistance between the solid flux and the mould and m is the heat transfer coefficient linking the temperature at outer surface of the solid flux and *T*_w_. The form of equation (3.8) is thus similar to that for Newton’s law of cooling, and is derived by considering one-dimensional heat transfer from the cold water stream, through a thermal boundary at the pipe surface and through the mould to the surface at *x*=0; the analysis is analogous to that in [16], pp. 1621–1622. Equation (3.8) is appropriate if there is a layer of solid flux at the mould wall; however, it is possible that during the oscillation cycle the temperature is high enough that there is no flux layer in solid phase, but rather in liquid phase; in this case, (3.8) would need to be replaced by

3.9 $$k_{f}^{\left(l\right)}\frac{\partial T}{\partial x}=m(T-T_{w});$$

here, the interface thermal contact resistance has been removed, since one can expect perfect contact between molten flux and the mould wall. A still further modification of this boundary condition would be for the situation when there is no flux at all between the mould wall and the solidified steel shell. This case appears to occur in [8] and, since it is the situation of poor solid–solid contact as was the case in equation (3.8), would be best represented by

3.10 $$k_{s}^{\left(s\right)}\frac{\partial T}{\partial x}=\frac{m}{1+mR_{\mathrm{ms}}}(T-T_{w}),$$

where *R*_ms_ is the interface thermal contact resistance between the solid steel and the mould; there would be no reason to expect *R*_ms_=*R*_mf_, but we will, for simplicity, take *R*_ms_=*R*_mf_=*R*. A further important quantity is the temperature at the outer surface of the mould, *T*_mould_, which can be determined after *T* is found, as

3.11 $$T_{\mathrm{mould}}=\frac{{\left(T\right)}_{x=0}+mRT_{w}}{1+mR}.$$

Similarly, we have the heat flux at this surface, *q*, which is defined by

3.12 $$q=\left\{ \begin{array}{ll} {\left(-k_{f}^{\left(s\right)}\frac{\partial T}{\partial x}\right)}_{x=0}, & \text{if }{\left(T\right)}_{x=0}<T_{m,f},\\ {\left(-k_{f}^{\left(l\right)}\frac{\partial T}{\partial x}\right)}_{x=0}, & \text{if }T_{m,f}\leq {\left(T\right)}_{x=0}\text{ and }s=0,\;h>0,\\ {\left(-k_{s}^{\left(s\right)}\frac{\partial T}{\partial x}\right)}_{x=0}, & \text{if }T_{m,f}\leq {\left(T\right)}_{x=0}\text{ and }s=0,\;h=0. \end{array}\right..$$

At *x*=s(*z*,*t*), we have

3.13 $$\begin{array}{rl} u_{z} & =V(t), \end{array}$$

3.14 $$\begin{array}{rl} u_{x} & =\frac{\partial s}{\partial t}+u_{z}\frac{\partial s}{\partial z}, \end{array}$$

3.15 $$\begin{array}{rl} T & =T_{m,f} \end{array}$$

3.16 $$\begin{array}{rl} \text{and}\;k_{f}^{\left(s\right)}{\left(\frac{\partial T}{\partial x}\right)}_{-}-k_{f}^{\left(l\right)}{\left(\frac{\partial T}{\partial x}\right)}_{+} & =\rho_{f}^{\left(l\right)}\Delta H_{f}\left(\frac{\partial s}{\partial t}+V\frac{\partial s}{\partial z}\right), \end{array}$$

where ( )_±_ denotes the value of a function in the limit as *x* tends to s(*z*,*t*) from above and below, respectively, and Δ*H*_f_ is the latent heat of fusion for the flux. Physically, equations (3.13)–(3.16) represent, respectively: the continuity of the *z*-component of velocity, so that the molten flux moves with the speed of the solid flux, which is in turn assumed to move with the speed of the mould wall, i.e. no slip; the continuity of the *x*-component of the velocity; the temperature is equal to that of the melting temperature of the flux; conservation of heat, relating the differences in heat flux to the latent heat released due to phase change. Since the effect of latent heat release is believed to be small [8], and it is seldom, if ever, included in others’ models for oscillation-mark formation, we will henceforth simply set the right-hand side of equation (3.16) to zero.

At *x*=s(*z*,*t*)+*h*(*z*,*t*), we have

3.17 $$\begin{array}{rl} & u_{z}=V_{\mathrm{cast}}, \end{array}$$

3.18 $$\begin{array}{rl} & \frac{\partial }{\partial t}(s+h)+u_{z}\frac{\partial }{\partial z}(s+h)=0, \end{array}$$

3.19 $$\begin{array}{rl} & {\left[T\right]}_{-}^{+}=0 \end{array}$$

3.20 $$\begin{array}{rl} \text{and}\; & k_{s}^{\left(s\right)}{\left(\frac{\partial T}{\partial x}\right)}_{+}=k_{f}^{\left(l\right)}{\left(\frac{\partial T}{\partial x}\right)}_{-}. \end{array}$$

The physical interpretations of (3.17)–(3.20) are, respectively: the continuity of the *z*-component of velocity, so that the molten flux moves with the speed of the solid steel; the *x*-component of the velocity is zero; the temperature is continuous; the heat flux is continuous.

At *x*=s(*z*,*t*)+*h*(*z*,*t*)+f(*z*,*t*),

3.21 $$T=T_{m,s}$$

and

3.22 $$k_{s}^{\left(s\right)}{\left(\frac{\partial T}{\partial x}\right)}_{-}-k_{s}^{\left(l\right)}{\left(\frac{\partial T}{\partial x}\right)}_{+}=\rho_{s}^{\left(l\right)}\Delta H_{s}\left(\frac{\partial }{\partial t}(s+h+f)+V_{\mathrm{cast}}\frac{\partial }{\partial z}(s+h+f)\right),$$

where Δ*H*_s_ is the latent heat of fusion for steel; note that using (3.17) and (3.18) reduces (3.22) to

3.23 $$k_{s}^{\left(s\right)}{\left(\frac{\partial T}{\partial x}\right)}_{-}-k_{s}^{\left(l\right)}{\left(\frac{\partial T}{\partial x}\right)}_{+}=\rho_{s}^{\left(l\right)}\Delta H_{s}\left(\frac{\partial f}{\partial t}+V_{\mathrm{cast}}\frac{\partial f}{\partial z}\right).$$

The physical meaning of (3.21) and (3.22) can be deduced from the previous discussion, and is therefore not repeated. However, we note that there is no governing equation to determine (∂*T*/∂*x*)_+_ in equation (3.23), and we will therefore set, for the time being,

3.24 $$Q(z,t):=k_{s}^{\left(l\right)}{\left(\frac{\partial T}{\partial x}\right)}_{+}$$

and consider the effect of this term later; in this respect, our approach is different from that in [8], where the temperature in the molten steel was calculated explicitly.

For the pressure, we have

3.25 $$p=p_{a}+\rho_{s}^{\left(l\right)}gz_{0}\;\text{at }z=z_{0}(t)$$

and

3.26 $$p=p_{a}\;\text{at }z=L.$$

The first of these is based on the idea that since molten flux and steel are in contact at *z*=*z*_0_(*t*), their pressures are equal [9]; the right-hand side of (3.25) is the molten steel metallostatic pressure, with *p*_*a*_ as the atmospheric pressure. On the other hand, equation (3.27) represents the fact that at some distance l down the caster, the pressure will once again be atmospheric, either because an air gap will form between the solidified flux and the mould, or at the bottom of the mould itself, where the system is exposed to the ambient atmosphere. For simplicity, we will take l to be the length of the mould, since we can then prescribe it; otherwise, its value would need to be determined; even so, it would still be of the order of magnitude of the length of the mould [9].

We can also note at this point that the interfacial conditions (3.13), (3.14), (3.17) and (3.18), in combination with equation (3.3), lead to

3.27 $$V_{\mathrm{cast}}\frac{\partial }{\partial z}(s+h)=\frac{\partial }{\partial z}\left(Vs+\int_{0}^{h(z,t)}u_{z}(s+\zeta ,t)\;d\zeta \right)$$

and thus, integrating with respect to *z*, as in [8],

3.28 $$V_{\mathrm{cast}}(s+h)-Vs+\int_{0}^{h(z,t)}u_{z}(s+\zeta ,t)\;d\zeta =Q_{R}(t),$$

where *Q*_*R*_ is a function of time that will have to be determined.

While (3.8)–(3.27) can be considered as boundary conditions for the lower zone, boundary conditions are also required for the upper zone and in particular for equation (3.7). For these, we have

3.29 $$\begin{array}{rl} p & =p_{a}\;\text{at }z=0, \end{array}$$

3.30 $$\begin{array}{rl} u_{z} & =V(t)\;\text{at }x=s_{0}(t) \end{array}$$

3.31 $$\begin{array}{rl} \text{and}\;\frac{\partial u_{z}}{\partial x} & =0\;\text{at }x=s_{0}(t)+h_{0}(t). \end{array}$$

Respectively, these express the following:

— the meniscus is at atmospheric pressure. The slag bed on top of the meniscus will be no more than a centimetre in height as seen, for example, in the detailed computations of Ramirez-Lopez *et al.* [7], and will, therefore, contribute a pressure head no greater that 2930×10×0.01≈293 *Pa*. Compared with the atmospheric pressure, 10^5^ *Pa*, this is clearly negligible;
— the continuity of the *z*-component of velocity, as in equation (3.13);
— zero shear stress at the interface of molten flux and molten steel, which arises from assuming continuity of shear stress at this interface, combined with the fact that the flux is much more viscous than the steel, giving the proposed simplification.

Note also that, in the original development, the momentum equations are solved for both upper and lower zones, whereas the heat equations are solved only in the lower zone; consequently, a boundary condition would be required at *z*=*z*_0_(*t*) for the temperature and, in [8], this is calculated to be the profile obtained by assuming a conductive temperature profile in the flux and steel layers.

The problem also formally requires initial conditions, but since we will be considering the periodic behaviour of the system that is eventually established, and since our treatment here is analytical rather than numerical, there is no need to specify these explicitly here.

## 4. Non-dimensionalization

Rather than attempting a numerical simulation to the above equations, we non-dimensionalize the model, with a view to identifying the key dimensionless parameters; this is so as to be able to propose a reduced model later on. For this purpose, we set

$$\begin{array}{rl} X & =\frac{x}{\left[x\right]},\;Z=\frac{z}{\left[z\right]},\;\tau =\frac{t}{\left[t\right]},\;U_{X}=\frac{u_{x}}{\left[x]V_{\mathrm{cast}}/[z\right]},\;U_{Z}=\frac{u_{z}}{V_{\mathrm{cast}}},\;\theta =\frac{T-T_{w}}{\Delta T},\\ F & =\frac{f}{\left[x\right]},\;H=\frac{h}{\left[x\right]},\;S=\frac{s}{\left[x\right]},\;P=\frac{p-p_{a}}{[\mu_{f}][z]V_{\mathrm{cast}}/{\left[x\right]}^{2}},\;{\bar{\mu }}_{f}=\frac{\mu_{f}}{\left[\mu_{f}\right]}\;\mathrm{and}\;Q=\frac{Q}{t[Q]}, \end{array}$$

where [*x*],[*z*],[*t*] and Δ*T* are, respectively, *x*-length, *z*-length, time and temperature difference scales which are taken to be

$$[x]={\left(\frac{[t]k_{f}^{\left(s\right)}}{\rho_{f}^{\left(s\right)}c_{f}^{\left(s\right)}}\right)}^{1/2},\;[t]=\frac{2\pi }{\omega },\;[z]=V_{\mathrm{cast}}[t],\;\Delta T=T_{m,s}-T_{w};$$

also, [*Q*] is a heat flux scale that we assume known. The numerical values for all model parameters are as given in table 1; these consist of the original data from Hill *et al.* [8] and more recent data, where different, from Saleem [13].

In the next §4.1, we give the non-dimensionalized governing equations and thereafter, in §4.2, the non-dimensionalized boundary and initial conditions. After that, in §4.3, the characteristic values of the model dimensionless parameters are given.

### 4.1. Governing equations

So, for 0<*X*<*S*(*Z*,*τ*), equation (3.1) gives

4.1 $$\frac{\partial \theta }{\partial \tau }+V\frac{\partial \theta }{\partial Z}=\frac{\partial^{2}\theta }{\partial X^{2}},$$

where

4.2 $$V=V_{0}\cos2\pi \tau ,$$

with $V_{0}=V_{0}/V_{\mathrm{cast}}.$

For *S*(*Z*,*τ*)<*X*<*S*(*Z*,*τ*)+*H*(*Z*,*τ*), equations (3.3)–(3.5) give

4.3 $$\begin{array}{rl} & \frac{\partial U_{X}}{\partial X}+\frac{\partial U_{Z}}{\partial Z}=0, \end{array}$$

4.4 $$\begin{array}{rl} & 0=-\frac{\partial P}{\partial Z}+\frac{\partial }{\partial X}\left({\bar{\mu }}_{f}\frac{\partial U_{Z}}{\partial X}\right)+\Lambda \end{array}$$

4.5 $$\begin{array}{rl} \text{and}\; & \alpha_{f}\left(\frac{\partial \theta }{\partial \tau }+U_{X}\frac{\partial \theta }{\partial X}+U_{Z}\frac{\partial \theta }{\partial Z}\right)=\frac{\partial^{2}\theta }{\partial X^{2}}, \end{array}$$

where ∂*P*/∂*Z* is a function of *Z* and *τ*, and

$$\alpha_{f}=\frac{k_{f}^{\left(s\right)}/\rho_{f}^{\left(s\right)}c_{f}^{\left(s\right)}}{k_{f}^{\left(l\right)}/\rho_{f}^{\left(l\right)}c_{f}^{\left(l\right)}},\;\Lambda =\frac{\rho_{f}^{\left(l\right)}g{\left[x\right]}^{2}}{[\mu_{f}]V_{\mathrm{cast}}}.$$

From equation (4.4), we can verify the validity of using the lubrication approximation. For this, we require that *Re*([*x*]/[*z*])^2^≪1, where *Re* is the Reynolds number, given by

$$Re=\frac{\rho_{f}^{\left(l\right)}V_{\mathrm{cast}}[z]}{\left[\mu_{f}\right]}.$$

Using the data in table 1, we have *Re*([*x*]/[*z*])^2^∼0.02, as required.

For *S*(*Z*,*τ*)+*H*(*Z*,*τ*)<*X*<*S*(*Z*,*τ*)+*H*(*Z*,*τ*)+f(*Z*,*τ*), equation (3.6) gives

4.6 $$\alpha_{s}\left(\frac{\partial \theta }{\partial \tau }+\frac{\partial \theta }{\partial Z}\right)=\frac{\partial^{2}\theta }{\partial X^{2}},$$

where

$$\alpha_{s}=\frac{k_{f}^{\left(s\right)}/\rho_{f}^{\left(s\right)}c_{f}^{\left(s\right)}}{k_{s}^{\left(l\right)}/\rho_{s}^{\left(l\right)}c_{s}^{\left(l\right)}}.$$

In addition to the above, equation (3.29) gives

4.7 $$(V-1)S+\int_{0}^{H(Z,\tau )}U_{Z}(S+\zeta ,\tau )\;d\zeta -H=Q_{R}^{*}(\tau ),$$

where $Q_{R}^{*}(\tau )=Q_{R}(\tau )/V_{\mathrm{cast}}[x].$

### 4.2. Boundary conditions

At *X*=0, if solid flux contacts the mould, we have

4.8 $$\frac{\partial \theta }{\partial X}=Bi_{\mathrm{ms}}\theta ,$$

where *Bi*_ms_ is the Biot number and is given by $Bi_{\mathrm{ms}}=m[x]/k_{f}^{\left(s\right)}(1+Rm).$ If liquid flux contacts the mould, then

4.9 $$\kappa \frac{\partial \theta }{\partial X}=Bi_{\mathrm{ml}}\theta ,$$

where $\kappa =k_{f}^{\left(l\right)}/k_{f}^{\left(s\right)}$ and $Bi_{\mathrm{ml}}=m[x]/k_{f}^{\left(s\right)}.$ If solid steel contacts the mould, then

4.10 $$\frac{\kappa }{K_{\mathrm{fs}}}\frac{\partial \theta }{\partial X}=Bi_{\mathrm{ms}}\theta ,$$

where $K_{\mathrm{fs}}=k_{f}^{\left(l\right)}/k_{s}^{\left(s\right)}.$

At *X*=*S*(*Z*,*τ*),

4.11 $$\begin{array}{rl} U_{Z} & =V, \end{array}$$

4.12 $$\begin{array}{rl} U_{X} & =\frac{\partial S}{\partial \tau }+U_{Z}\frac{\partial S}{\partial Z}, \end{array}$$

4.13 $$\begin{array}{rl} \theta & =\theta_{m,f} \end{array}$$

4.14 $$\begin{array}{rl} \text{and}\;{\left(\frac{\partial \theta }{\partial X}\right)}_{-} & =\kappa {\left(\frac{\partial \theta }{\partial X}\right)}_{+}, \end{array}$$

where *θ*_m,f_=(*T*_m,f_−*T*_w_)/(*T*_m,s_−*T*_w_).

At *X*=*S*(*Z*,*τ*)+*H*(*Z*,*τ*), we have

4.15 $$\begin{array}{rl} & U_{Z}=1, \end{array}$$

4.16 $$\begin{array}{rl} & \frac{\partial }{\partial \tau }(S+H)+\frac{\partial }{\partial Z}(S+H)=0, \end{array}$$

4.17 $$\begin{array}{rl} & {\left[\theta \right]}_{-}^{+}=0 \end{array}$$

4.18 $$\begin{array}{rl} \text{and}\; & {\left(\frac{\partial \theta }{\partial X}\right)}_{+}=K_{\mathrm{fs}}{\left(\frac{\partial \theta }{\partial X}\right)}_{-}. \end{array}$$

At *X*=*S*(*Z*,*τ*)+*H*(*Z*,*τ*)+f(*Z*,*τ*), we have

4.19 θ=1

and

4.20 $${\left(\frac{\partial \theta }{\partial X}\right)}_{-}-KQ(Z,\tau )=\frac{1}{St_{s}}\left(\frac{\partial F}{\partial \tau }+\frac{\partial F}{\partial Z}\right),$$

where

$$St_{s}=\frac{k_{s}^{\left(s\right)}[t]\Delta T}{\rho_{s}^{\left(l\right)}\Delta H_{s}{\left[x\right]}^{2}},\;K=\frac{\left[Q][x\right]}{k_{s}^{\left(s\right)}\Delta T}.$$

For *P*, we have

4.21 $$P=\Gamma Z_{0}\;\text{at }Z=Z_{0}(\tau )$$

and

4.22 $$P=0\;\text{at }Z=Z_{L},$$

where

$$\Gamma =\frac{\rho_{s}g{\left[x\right]}^{2}}{[\mu_{f}]V_{\mathrm{cast}}},\;Z_{L}=\frac{L}{V_{\mathrm{cast}}[t]}.$$

### 4.3. Non-dimensional parameters

Taking, for simplicity, *Bi*_ml_=*Bi*_ms_=*Bi*, there are, by this stage, 12 non-dimensional parameters:

$$Bi,K_{\mathrm{fs}},K\text{,}\;St_{s},V_{0},Z_{L},\alpha_{f},\alpha_{s},\Gamma ,\kappa ,\Lambda ,\theta_{m,f}.$$

The values of the eleven of these corresponding to the datasets of [8] and [13], which will be use later in §7, are given in table 2; the likely value of the missing one, K, is discussed in §6.1.

**Table 2.** Values of dimensionless parameters using data from Hill *et al.* [8] and Saleem [13].

	Hill *et al.*[8]	Saleem [13]
*Bi*	1.74	1.06
*K*_fs_	0.075	0.075
*St*_s_	51.3	51.3
$V_{0}$	2.79	2.22
*Z*_l_	104	85.3
*α*_f_	0.667	0.667
*α*_s_	0.071	0.071
*Γ*	20.7	21.6
*κ*	1.5	1.5
*Λ*	7.79	8.10
*θ*_m,f_	0.723	0.723

Moreover, for slender geometry approximation to be valid, we also require that [*x*]/[*z*]≪1; from the parameters in table 1, we find that [*x*]/[*z*]∼0.1, so that the approximation is suitably valid.

## 5. Reduced model

We now proceed by proposing a reduced model by making use of the sizes of the dimensionless parameters to simplify the governing equations, as appropriate. In view of the values given in §4.3, we observe that

$$K_{\mathrm{fs}},\alpha_{s}\ll 1,\;St_{s},Z_{L}\gg 1,\;Bi,V_{0},\alpha_{f},\Gamma ,\kappa ,\Lambda ,\theta_{m,f}∼O(1);$$

thence, we arrive at the following reduced model equations.

### 5.1. Governing equations

So, for 0<*X*<*S*(*Z*,*τ*),

5.1 $$\frac{\partial \theta }{\partial \tau }+V\frac{\partial \theta }{\partial Z}=\frac{\partial^{2}\theta }{\partial X^{2}}.$$

For *S*(*Z*,*τ*)<*X*<*S*(*Z*,*τ*)+*H*(*Z*,*τ*),

5.2 $$\begin{array}{rl} & \frac{\partial U_{X}}{\partial X}+\frac{\partial U_{Z}}{\partial Z}=0, \end{array}$$

5.3 $$\begin{array}{rl} & 0=-\frac{\partial P}{\partial Z}+\frac{\partial }{\partial X}\left({\bar{\mu }}_{f}\frac{\partial U_{Z}}{\partial X}\right)+\Lambda \end{array}$$

5.4 $$\begin{array}{rl} \text{and}\; & \alpha_{f}\left(\frac{\partial \theta }{\partial \tau }+U_{X}\frac{\partial \theta }{\partial X}+U_{Z}\frac{\partial \theta }{\partial Z}\right)=\frac{\partial^{2}\theta }{\partial X^{2}}. \end{array}$$

For *S*(*Z*,*τ*)+*H*(*Z*,*τ*)<*X*<*S*(*Z*,*τ*)+*H*(*Z*,*τ*)+f(*Z*,*τ*),

5.5 $$0=\frac{\partial^{2}\theta }{\partial X^{2}}.$$

In addition to the above, equation (4.7) remains unchanged.

### 5.2. Boundary conditions

At *X*=0, (4.8)–(4.10) become

5.6 $$\begin{array}{rl} \frac{\partial \theta }{\partial X} & =Bi\;\theta , \end{array}$$

5.7 $$\begin{array}{rl} \kappa \frac{\partial \theta }{\partial X} & =Bi\;\theta \end{array}$$

5.8 $$\begin{array}{rl} \text{and}\;\kappa \frac{\partial \theta }{\partial X} & =Bi\;K_{\mathrm{fs}}\theta , \end{array}$$

respectively; although *K*_fs_≪1, we will retain this because of the existence of large parameter, *St*_s_, as discussed presently.

At *X*=*S*(*Z*,*τ*),

5.9 $$\begin{array}{rl} U_{Z} & =V, \end{array}$$

5.10 $$\begin{array}{rl} U_{X} & =\frac{\partial S}{\partial \tau }+U_{Z}\frac{\partial S}{\partial Z}, \end{array}$$

5.11 $$\begin{array}{rl} \theta & =\theta_{m,f} \end{array}$$

5.12 $$\begin{array}{rl} \text{and}\;{\left(\frac{\partial \theta }{\partial X}\right)}_{-} & =\kappa {\left(\frac{\partial \theta }{\partial X}\right)}_{+}. \end{array}$$

At *X*=*S*(*Z*,*τ*)+*H*(*Z*,*τ*),

5.13 $$\begin{array}{rl} & U_{Z}=1, \end{array}$$

5.14 $$\begin{array}{rl} & \frac{\partial }{\partial \tau }(S+H)+\frac{\partial }{\partial Z}(S+H)=0, \end{array}$$

5.15 $$\begin{array}{rl} & {\left[\theta \right]}_{-}^{+}=0 \end{array}$$

5.16 $$\begin{array}{rl} \text{and}\; & {\left(\frac{\partial \theta }{\partial X}\right)}_{+}=0. \end{array}$$

At *X*=*S*(*Z*,*τ*)+*H*(*Z*,*τ*)+f(*Z*,*τ*),

5.17 θ=1

and

5.18 $${\left(\frac{\partial \theta }{\partial X}\right)}_{-}-KQ(Z,\tau )=\frac{1}{St_{s}}\left(\frac{\partial F}{\partial \tau }+\frac{\partial F}{\partial Z}\right).$$

## 6. Analysis

We proceed by analysing, in §6.1 and 6.2, the heat and momentum equations, respectively. The analytical forms found for the temperature and velocity field are then used, in §6.3, to constitute solutions for the actual oscillation-mark profile.

### 6.1. Heat

Considering the solid steel, the equations there have reduced to, on using *α*_s_,*K*_fs_≪1,*St*_s_≫1,

6.1 $$\frac{\partial^{2}\theta }{\partial X^{2}}=0,$$

subject to

6.2 $$\begin{array}{rl} \frac{\partial \theta }{\partial X} & =0\;\text{at }X=S(Z,\tau )+H(Z,\tau ), \end{array}$$

6.3 $$\begin{array}{rl} \theta & =1\;\text{at }X=S(Z,\tau )+H(Z,\tau )+F(Z,\tau ) \end{array}$$

6.4 $$\begin{array}{rl} \text{and}\;{\left(\frac{\partial \theta }{\partial X}\right)}_{-} & =KQ(Z,\tau )\;\text{at }X=S(Z,\tau )+H(Z,\tau )+F(Z,\tau ). \end{array}$$

If there is no or little superheat, the right-hand side of equation (6.4) can be set to zero, and we have just *θ*≡1 as the solution at leading order, i.e. the solid steel will be at the melting temperature. Now, since *St*_s_*K*_fs_∼*O*(1), it is appropriate to consider a regular perturbation expansion for *θ* in the solid steel region of the form

6.5 $$\theta =\theta^{\left(0\right)}+K_{\mathrm{fs}}\theta^{\left(1\right)}+O(K_{\mathrm{fs}}^{2}),$$

leading to *θ*^(0)^≡1 and

6.6 $$\frac{\partial^{2}\theta^{\left(1\right)}}{\partial X^{2}}=0,$$

subject to

6.7 $$\begin{array}{rl} \frac{\partial \theta^{\left(1\right)}}{\partial X} & ={\left(\frac{\partial \theta }{\partial X}\right)}_{-}\;\text{at }X=S(Z,\tau )+H(Z,\tau ), \end{array}$$

6.8 $$\begin{array}{rl} \theta^{\left(1\right)} & =0\;\text{at }X=S(Z,\tau )+H(Z,\tau )+F(Z,\tau ) \end{array}$$

6.9 $$\begin{array}{rl} \text{and}\;{\left(\frac{\partial \theta^{\left(1\right)}}{\partial X}\right)}_{-}-\varphi Q(Z,\tau ) & =\lambda \left(\frac{\partial F}{\partial \tau }+\frac{\partial F}{\partial Z}\right)\;\text{at }X=S(Z,\tau )+H(Z,\tau )+F(Z,\tau ), \end{array}$$

where *λ*=1/*St*_s_*K*_fs_, $\varphi =K/K_{\mathrm{fs}}∼O(1)$ and Q=Q/[Q]; note that the idea of combining two dimensionless parameters, one of which is large (1/*K*_fs_) and the other is small (1/*St*_s_) but whose product is *O*(1), into one *O*(1) parameter was used in a similar way recently in an asymptotic model for phase change in pharmaceutical lyophilization [18]. Note also that, for equation (6.9) to constitute a sensible balance, we can at most have that *φ*∼*O*(1), indicating that $K\leq K_{\mathrm{fs}}$. Furthermore, for consistency, we should also expand all of the other dependent variables in terms of *K*_fs_, but we suppress the superscript notation, ^( )^, used in (6.5), on the understanding that it is the leading-order solution for f,*H*,*S* and *θ* in the solid and molten flux region that we are finding. So, we have

6.10 $$\theta^{\left(1\right)}=A(Z,\tau )\{X-S(Z,\tau )-H(Z,\tau )-F(Z,\tau )\},$$

where

6.11 $$A(Z,\tau )=\lambda \left(\frac{\partial F}{\partial \tau }+\frac{\partial F}{\partial Z}\right)+\varphi Q(Z,\tau ).$$

Thus, we have eliminated the solid steel region, in the sense that *θ* there can be found after *S*,f and *H* have been determined. Moreover, the remaining problem is now posed on just 0<*X*<*S*(*Z*,*τ*)+*H*(*Z*,*τ*), with the boundary conditions at *X*=*S*(*Z*,*τ*)+*H*(*Z*,*τ*) being

6.12 θ=1

and

6.13 $$\frac{\partial \theta }{\partial X}=\lambda \left(\frac{\partial F}{\partial \tau }+\frac{\partial F}{\partial Z}\right)+\varphi Q(Z,\tau ).$$

Next, we have, for the molten flux region, if we assume *α*_f_≪1,

6.14 $$\theta =1+\left\{\lambda \left(\frac{\partial F}{\partial \tau }+\frac{\partial F}{\partial Z}\right)+\varphi Q(Z,\tau )\right\}(X-S-H).$$

Moreover, if we assume that that we can neglect the left-hand side in (5.1), we obtain, for the solid flux region,

6.15 $$\theta =\theta_{m,f}\left(\frac{Bi\;X+1}{Bi\;S+1}\right).$$

Also, we will have

6.16 $$\theta_{m,f}=1-\left\{\lambda \left(\frac{\partial F}{\partial \tau }+\frac{\partial F}{\partial Z}\right)+\varphi Q(Z,\tau )\right\}H,$$

as well as a partial differential equation relating *S* and f,

6.17 $$\frac{Bi\;\theta_{m,f}}{Bi\;S+1}=\kappa \left\{\lambda \left(\frac{\partial F}{\partial \tau }+\frac{\partial F}{\partial Z}\right)+\varphi Q(Z,\tau )\right\}.$$

Thus, combining (6.16) and (6.17), we find

6.18 $$\frac{Bi\;\theta_{m,f}}{\kappa \lambda (Bi\;S+1)}=\frac{1-\theta_{m,f}}{\lambda H},$$

so that *S* and *H* are related by

6.19 $$H=\kappa \left(\frac{1}{\theta_{m,f}}-1\right)\left(S+\frac{1}{Bi}\right).$$

Hence, if we can find *S*, we will automatically have *H* and f.

We also need the corresponding result when there is no solid flux. In this case, we have just

6.20 $$\theta =\frac{Bi\;X+\kappa }{Bi\;H+\kappa }$$

for the molten flux region, which is consistent provided that

$$\frac{\kappa }{Bi\;H+\kappa }\geq \theta_{m,f},$$

i.e.

$$H\leq \frac{1}{Bi(C-1/2)},\;\text{where}\;C=\frac{\theta_{m,f}}{\kappa (1-\theta_{m,f})}+\frac{1}{2}.$$

Also, instead of equation (6.17), we have

6.21 $$\frac{Bi}{Bi\;H+\kappa }=\lambda \left(\frac{\partial F}{\partial \tau }+\frac{\partial F}{\partial Z}\right)+\varphi Q(Z,\tau ).$$

Defining *σ*:=*S*+*H*, we have

6.22 $$\sigma =H+\max\left(\left(C-\frac{1}{2}\right)H-\frac{1}{Bi},0\right).$$

If there is no flux present at all, we have (6.6), subject to

6.23 $$\begin{array}{rl} \kappa \frac{\partial \theta^{\left(1\right)}}{\partial X} & =Bi\;\text{at }X=0, \end{array}$$

6.24 $$\begin{array}{rl} \theta^{\left(1\right)} & =0\;\text{at }X=F(Z,\tau ) \end{array}$$

6.25 $$\begin{array}{rl} \text{and}\;{\left(\frac{\partial \theta^{\left(1\right)}}{\partial X}\right)}_{-} & =\lambda \left(\frac{\partial F}{\partial \tau }+\frac{\partial F}{\partial Z}\right)+\varphi Q(Z,\tau )\;\text{at }X=F(Z,\tau ). \end{array}$$

Thus, we have

6.26 $$\theta^{\left(1\right)}=\frac{Bi}{\kappa }(X-F),$$

where f satisfies

6.27 $$\frac{Bi}{\kappa }=\lambda \left(\frac{\partial F}{\partial \tau }+\frac{\partial F}{\partial Z}\right)+\varphi Q(Z,\tau ).$$

### 6.2. Momentum

#### 6.2.1. Lower zone

Consider now ${\bar{\mu }}_{f}=1,$ leading to

6.28 $$\frac{\partial^{2}U_{Z}}{\partial X^{2}}=\frac{\partial \bar{P}}{\partial Z},$$

where $\bar{P}=P-\Lambda Z.$ Since the right-hand side is a function of *Z* and *τ*, we have

6.29 $$U_{Z}=\frac{1}{2}\Pi (Z,\tau )X^{2}+F_{1}(Z,\tau )X+F_{2}(Z,\tau ),$$

with $\Pi :=\partial \bar{P}/\partial Z,$ where f_1_ and f_2_ are functions to be determined.

Also, the dimensionless liquid flux, *Q*^(l)^_l_, is given by

$$Q_{L}^{\left(l\right)}=\int_{S}^{S+H}U_{Z}(\xi ,\tau )\;d\xi .$$

Now, we have

6.30 $$\frac{\partial \sigma }{\partial \tau }+\frac{\partial \sigma }{\partial Z}=0$$

and

6.31 $$\frac{\partial \sigma }{\partial \tau }+\frac{\partial }{\partial Z}\left(VS+\frac{1}{6}\Pi (Z,\tau )(\sigma^{3}-S^{3})+\frac{1}{2}F_{1}(Z,\tau )(\sigma^{2}-S^{2})+F_{2}(Z,\tau )(\sigma -S)\right)=0,$$

where $F_{1}^{L}$ and $F_{2}^{L}$ are given by

6.32 $$\left(\sigma -S)F_{1}(Z,\tau )=1-V-\frac{1}{2}\Pi (Z,\tau )(\sigma^{2}-S^{2}\right)$$

and

6.33 $$(\sigma -S)F_{2}(Z,\tau )=V\sigma -S+\frac{\sigma S}{2}\Pi (Z,\tau )(\sigma -S).$$

Hence,

6.34 $$(V-1)\frac{\partial \sigma }{\partial Z}=\frac{\partial }{\partial Z}\left(\frac{1}{2}(V-1)H+\frac{1}{12}H^{3}\Pi (Z,\tau )\right),$$

note that *Q*^(l)^_l_ is given by

6.35 $$Q_{L}^{\left(l\right)}=H(\frac{1}{2}(V+1)-\frac{1}{12}H^{2}\Pi (Z,\tau )).$$

Hence, integrating (6.34) with respect to *Z*, we obtain

6.36 $$(V-1)\left(S+\frac{H}{2}\right)-\frac{1}{12}H^{3}\Pi (Z,\tau )=Q_{R}^{*}(\tau ).$$

$Q_{R}^{*}(\tau )$ can be determined from boundary conditions (4.21) and (4.22). We find

$$\int_{Z_{0}(\tau )}^{Z_{L}}\Pi (Z,\tau )\;dZ=12(V-1)\int_{Z_{0}(\tau )}^{Z_{L}}\left(S+\frac{H}{2}\right)\frac{dZ}{H^{3}}-12Q_{R}^{*}(\tau )\int_{Z_{0}(\tau )}^{Z_{L}}\frac{dZ}{H^{3}},$$

leading to

6.37 $$-\Gamma Z_{0}(\tau )-\Lambda (Z_{L}-Z_{0}(\tau ))=12(V-1)\int_{Z_{0}(\tau )}^{Z_{L}}\left(S+\frac{H}{2}\right)\frac{dZ}{H^{3}}-12Q_{R}^{*}(\tau )\int_{Z_{0}(\tau )}^{Z_{L}}\frac{dZ}{H^{3}}.$$

Note that *Z*_0_(*τ*)≤1 and *Z*_l_≫1, and that *Γ*∼*Λ*, indicating that equation (6.37) can be reduced to

$$12(V-1)\int_{0}^{Z_{L}}\left(S+\frac{H}{2}\right)\frac{dZ}{H^{3}}-12Q_{R}^{*}(\tau )\int_{0}^{Z_{L}}\frac{dZ}{H^{3}}\approx -\Lambda Z_{L},$$

so that

6.38 $$Q_{R}^{*}(\tau )=\frac{12C(V-1)\int_{0}^{Z_{L}}(S+H/2)(dZ/H^{3})+\Lambda Z_{L}}{12\int_{0}^{Z_{L}}(dZ/H^{3})},$$

i.e. the unknown *Z*_0_(*τ*) is eliminated. Although $Q_{l}^{L}$ is given by (6.35), it is not in a convenient form, since *Π*(*Z*,*τ*) is not known. However, since

$$(V-1)S+Q_{L}^{\left(l\right)}-H=Q_{R}^{*}(\tau ),$$

we have

6.39 $$Q_{L}^{\left(l\right)}=\frac{12C(V-1)\int_{0}^{Z_{L}}(S+H/2)(dZ/H^{3})+\Lambda Z_{L}}{12\int_{0}^{Z_{L}}(dZ/H^{3})}+H-(V-1)S,$$

so that *Π* would be given by

6.40 $$\Pi (Z,\tau )=\frac{12}{H^{3}}\left\{(V-1)\left(S+\frac{H}{2}\right)-\frac{12C(V-1)\int_{0}^{Z_{L}}(S+H/2)(dZ/H^{3})+\Lambda Z_{L}}{12\int_{0}^{Z_{L}}(dZ/H^{3})}\right\}.$$

#### 6.2.2. Upper zone

In the upper zone, we have

6.41 $$\frac{\partial^{2}U_{Z}}{\partial X^{2}}=\frac{\partial P}{\partial Z}-\Lambda ,$$

with

6.42 P=0at Z=0

and

6.43 $$P=\Gamma Z_{0}\;\text{at }Z=Z_{0}(\tau ),$$

leading to

6.44 $$U_{Z}=\frac{1}{2}(\Gamma -\Lambda )X^{2}+F_{3}(Z,\tau )X+F_{4}(Z,\tau ),$$

where

6.45 $$U_{Z}=V\;\text{at }X=S$$

and

6.46 $$\frac{\partial U_{Z}}{\partial X}=0\;\text{at }X=S+H$$

and with f_3_ and f_4_ functions to be determined. So,

6.47 $$F_{1}^{U}=-(\Gamma -\Lambda )\sigma ,\;F_{2}^{U}=V+\frac{1}{2}(\Gamma -\Lambda )S(2\sigma -S),$$

so that the dimensionless liquid flux in the upper zone, *Q*^(l)^_*U*_, is given by

6.48 $$Q_{U}^{\left(l\right)}=VH-\frac{1}{3}(\Gamma -\Lambda )H^{3}.$$

Now, we need

6.49 $$Q_{L}^{\left(l\right)}=Q_{U}^{\left(l\right)}\;\text{at }Z=Z_{0}(\tau ),$$

which gives

6.50 $$\frac{1}{3}(\Gamma -\Lambda )H^{3}-(V-1)(S+H)+\frac{12C(V-1)\int_{0}^{Z_{L}}(S+H/2)(dZ/H^{3})+\Lambda Z_{L}}{12\int_{0}^{Z_{L}}(dZ/H^{3})}=0.$$

Also, in view of (6.30), we must have

$$S(Z,\tau )+H(Z,\tau )=S_{0}(\tau -Z)+H_{0}(\tau -Z),$$

whence (6.19) implies that

$$S(Z,\tau )=S_{0}(\tau -Z),\;H(Z,\tau )=H_{0}(\tau -Z).$$

So, on setting *ζ*=*τ*−*Z*, we have

6.51 $$\frac{1}{3}(\Gamma -\Lambda )H_{0}^{3}-(V-1)(S_{0}+H_{0})+\frac{12C(V-1)\int_{\tau -Z_{L}}^{\tau }(S_{0}+H_{0}/2)(dZ/H_{0}^{3})+\Lambda Z_{L}}{12\int_{\tau -Z_{L}}^{\tau }(dZ/H_{0}^{3})}=0.$$

Recalling that

6.52 $$S_{0}=\left\{ \begin{array}{ll} 0 & \text{if }H_{0}\leq H_{\mathrm{crit}}\\ \frac{\theta_{m,f}H_{0}}{\kappa (1-\theta_{m,f})}-\frac{1}{Bi} & \text{if }H_{0}>H_{\mathrm{crit}} \end{array}\right.,$$

where $H_{\mathrm{crit}}=\kappa (1-\theta_{m,f})/Bi\;\theta_{m,f}=1/Bi(C-\frac{1}{2}),$ we see that (6.51) can be formulated just in terms of *H*_0_.

### 6.3. Solutions

We see that when V=1, we have

$$\frac{1}{3}(\Gamma -\Lambda )H_{0}^{3}+\frac{\Lambda Z_{L}}{12\int_{\tau -Z_{L}}^{\tau }(dZ/H_{0}^{3})}=0$$

and the only possibility is that *H*_0_=0 for that value of *τ*; call it τ*. However, we see that *H*_0_ must also have vanished at *τ*=τ*−1,τ*−2,… So, when V≠1, the integrals in (6.51) will be singular. It appears that the denominator of the last term on the left-hand side (6.51) is more singular than the numerator, since the most singular terms in the integrands in the numerator and denominator behave as $1/H_{0}^{2}$ and $1/H_{0}^{3},$ respectively; this suggests that the entire term can be neglected. We are left considering

6.53 $$\frac{1}{3}(\Gamma -\Lambda )H_{0}^{3}-(V-1)(S_{0}+H_{0})=0,$$

with *S*_0_ given by (6.52). The possible solutions are *H*_0_=0 if V≤1, and, if V>1,

6.54 $$H_{0}=0,\;\pm {\left(3\left(\frac{V-1}{\Gamma -\Lambda }\right)\right)}^{1/2}\;\text{if }H_{0}\leq H_{\mathrm{crit}}$$

and *H*_0_=H_*_ if *H*_0_>*H*_crit_, where H_*_ satisfies

6.55 $$\frac{1}{3}(\Gamma -\Lambda )H_{*}^{3}-(V-1)\left(\left(C+\frac{1}{2}\right)H_{*}-\frac{1}{Bi}\right)=0.$$

Putting

$$H_{*}=2{\left(C+\frac{1}{2}\right)}^{1/2}{\left(\frac{V-1}{\Gamma -\Lambda }\right)}^{1/2}\cos\chi ,$$

we use the well-known triple-angle identity,

$$\cos3\chi =4\cos^{3}\chi -3\cos\chi$$

to obtain

$$\cos3\chi +\frac{3{\left(\Gamma -\Lambda \right)}^{1/2}}{2\;Bi{\left(V-1\right)}^{1/2}{\left(C+1/2\right)}^{3/2}}=0,$$

whence

$$\chi =\frac{1}{3}\left\{2n\pi +\cos^{-1}\left(-\frac{3{\left(\Gamma -\Lambda \right)}^{1/2}}{2\;Bi{\left(C+1/2\right)}^{3/2}{\left(V-1\right)}^{1/2}}\right)\right\},$$

where *n* is an integer. The three roots can then be obtained by setting *n*=0,1,2; it turns out that *n*=1 gives the negative root, and *n*=0,2 give the positive roots, with *n*=0 giving the positive root for which H_*_>*H*_crit_.

Turning to the solution for f, equation (6.17) now gives

6.56 $$\kappa \left\{\lambda \left(\frac{\partial F}{\partial \tau }+\frac{\partial F}{\partial Z}\right)+\varphi Q(Z,\tau )\right\}=\frac{Bi\;\theta_{m,f}}{Bi\;S_{0}(\tau -Z)+1},$$

whereas (6.21) gives

6.57 $$\kappa \left\{\lambda \left(\frac{\partial F}{\partial \tau }+\frac{\partial F}{\partial Z}\right)+\varphi Q(Z,\tau )\right\}=\frac{\kappa \;Bi}{Bi\;H_{0}(\tau -Z)+\kappa }.$$

Setting

$$G(\tau -Z)=\left\{ \begin{array}{ll} \frac{Bi\;\theta_{m,f}}{Bi\;S_{0}(\tau -Z)+1} & \text{if }S_{0}>0,\\ \frac{\kappa \;Bi}{Bi\;H_{0}(\tau -Z)+\kappa } & \text{if }S_{0}=0,H_{0}>0,\\ Bi & \text{if }S_{0}=0,H_{0}=0, \end{array}\right.$$

we have

6.58 $$\kappa \left\{\lambda \left(\frac{\partial F}{\partial \tau }+\frac{\partial F}{\partial Z}\right)+\varphi Q(Z,\tau )\right\}=G(\tau -Z).$$

Putting

$$\hat{\tau }=\tau ,\;\zeta =\tau -Z,$$

equation (6.58) becomes

6.59 $$\kappa \left\{\lambda \frac{\partial F}{\partial \hat{\tau }}+\varphi Q(\zeta ,\hat{\tau })\right\}=G(\zeta )$$

and, in general, it would be necessary to know $Q(\zeta ,\hat{\tau })$ to make further analytical progress. However, it is instructive to consider the case of negligible superheat, since this will give us the upper limit for the solidified shell thickness. Setting the second term on the left-hand side of (6.59) to zero, we can integrate once with respect to $\hat{\tau }$ to give

$$F(\hat{\tau },\zeta )=\frac{1}{\kappa \lambda }(G(\zeta )\hat{\tau }+\tilde{F}(\zeta )),$$

where $\tilde{F}$ is a function to be determined, subject to

F=0at Z=0.

Thus, we have

6.60 $$\tilde{F}(\hat{\tau })=-\hat{\tau }G(\hat{\tau }),$$

leading to

6.61 $$F(Z,\tau )=\frac{Z}{\kappa \lambda }G(\tau -Z).$$

Finally, we note that, although it was stated at the outset in equation (3.2) that the mould velocity is a cosine function of time, the analysis has gone through without this fact having been used; consequently, the results obtained are also valid for non-sinusoidal mould oscillations.

## 7. Results

Here, we present results obtained with the reduced model. In §7.1, this is done using the original data in Hill *et al.* [8], whereas in §7.2, we give results using data from Saleem [13], as well as a comparison with his experimental results.

### 7.1. Data from Hill *et al.* [8]

**Figure 6.**
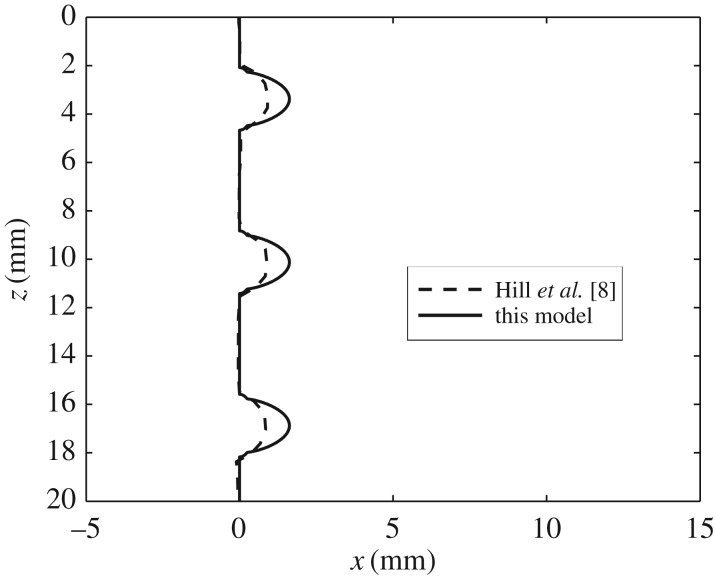
Comparison of the calculated geometry of oscillation marks on the steel surface, *s*+**h**, using data from Hill *et al.* [8].

Figure 6 shows the location of the oscillation marks on steel surface, *s*+*h*, as computed by our model and using model parameters from Hill *et al.* [8]; for comparison, the profile computed in [8] has also been added. It is evident that the results are both qualitatively similar, in that periodically spaced marks are obtained; thus, although we have treated the governing equations differently, we nevertheless obtain the pitch to be given by 2*πV*_cast_/*ω*. Although our model gives oscillation marks that are slightly deeper than those computed by Hill *et al.* [8], the maximum depth obtained is still within the 0.5–2 mm range of experimentally observed oscillation marks quoted in [8]. This maximum depth of the oscillation-mark is given in dimensional terms by

7.1 $${\left(\frac{2\pi k_{f}^{\left(s\right)}}{\omega \rho_{f}^{\left(s\right)}c_{f}^{\left(s\right)}}\right)}^{1/2}\left(\frac{k_{f}^{\left(s\right)}(T_{m,f}-T_{w})}{k_{f}^{\left(l\right)}(T_{m,s}-T_{m,f})}+1\right)H_{*}^{\max}-\frac{(1+Rm)k_{f}^{\left(s\right)}}{m},$$

where

$$H_{*}^{\max}=2{\left(C+\frac{1}{2}\right)}^{1/2}{\left(\frac{V_{0}-1}{\Gamma -\Lambda }\right)}^{1/2}\cos\left(\frac{1}{3}\cos^{-1}\left(-\frac{3{\left(\Gamma -\Lambda \right)}^{1/2}}{2\;Bi{\left(C+1/2\right)}^{3/2}{\left(V_{0}-1\right)}^{1/2}}\right)\right).$$

**Figure 7.**
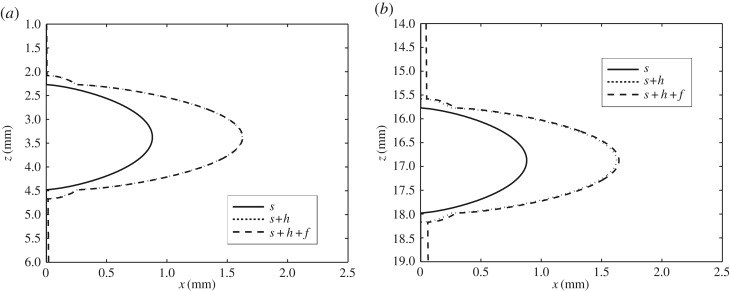
A blow-up of the region around the (*a*) first and (*b*) third oscillation marks, showing the molten flux/solid flux interface, s, the oscillation marks on the steel surface, *s*+*h*, and the steel solid–liquid interface, *s*+*h*+*f*, using data from Hill *et al.* [8].

Figures 7*a*,*b* show blow-ups of the regions around the first and third oscillation marks from the start of solidification, respectively. Here, there are several features of note. First, s and *s*+*h* are identical for both cases, as indicated in the analysis; however, *s*+*h*+f is greater in figure 7*b* than in figure 7*a*, as one would expect, since the solidified shell should be thicker the greater the distance from the meniscus. Note that the profiles here, as in §7.2, have been computed by setting *Q*=0 for simplicity, i.e. no superheat; observe also from the foregoing analysis that this simplification does not, at leading order, affect the profiles obtained for s and *s*+*h*, because *K*_fs_≪1.

### 7.2. Data from Saleem [13]

**Figure 8.**
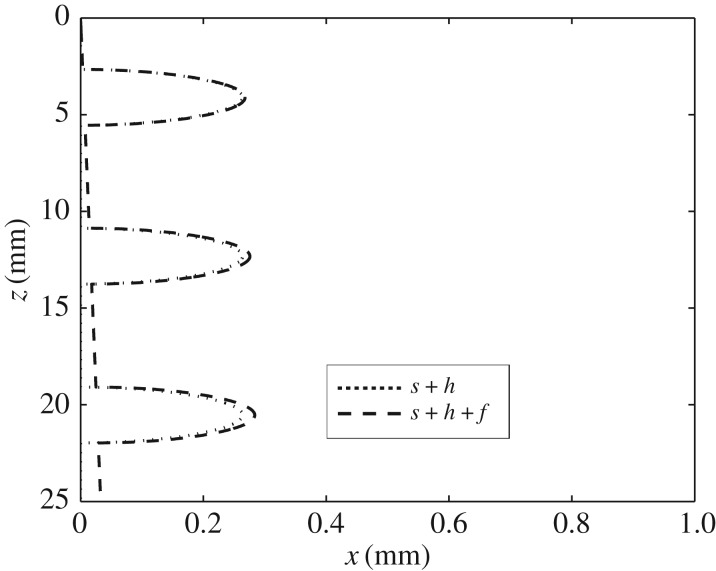
The calculated geometry of the oscillation marks on the steel surface, *s*+*h*, and the steel solid–liquid interface, *s*+*h*+*f*, using data from Saleem [13].

Figure 8 shows the resulting profiles for *s*+*h* and *s*+*h*+f, as computed by the model and using data from Saleem [13]; for the location of the oscillation marks, *s*+*h*, the result is qualitatively similar to that in figure 6, but the major difference is that the oscillation-mark depth is considerably smaller. Moreover, there is only molten flux and consequently there is no jump in the gradient of the oscillation-mark profile; as a result, the maximum depth of the oscillation-mark is given in dimensional terms by just

7.2 $${\left(\frac{3[\mu_{f}](V_{0}-V_{\mathrm{cast}})}{(\rho_{s}-\rho_{f})g}\right)}^{1/2}.$$

A surprising feature of this formula is that it contains neither *R* nor m, in contrast with (7.1). The interpretation is that neither of these quantities are of significance for oscillation marks that are sufficiently shallow. Also, as in figure 7*a*,*b*, we see that the thickness of the solidified shell increases with distance from the initial solidification point.

**Figure 9.**
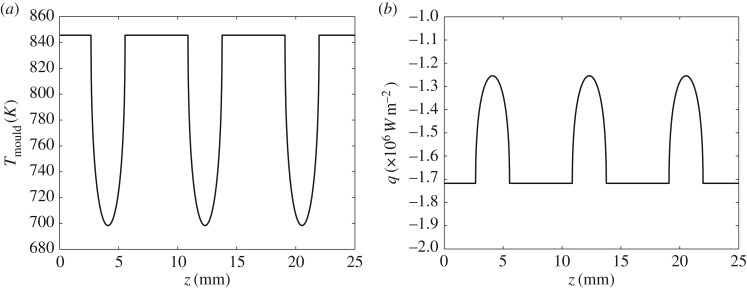
(*a*) Temperature at the mould wall, *T*_mould_; (*b*) heat flux at the mould wall, *q*.

Figure 9*a* shows the temperature at the surface of the mould wall, *T*_mould_, and corresponds to the oscillation-mark formation given in figure 8; figure 9*b* shows the corresponding heat flux *q*. Comparing the profiles in figure 9*a*,*b* with that in figure 8, we see that the oscillation mark forms at the same time as there is a decrease in the mould temperature at the mould surface and an increase in the heat flux. This is in line with the observations of Badri *et al.* [19,20], who showed that a sudden increase in heat flux must occur during the negative strip time, i.e. the time when the mould travels downwards faster than the strand. Note that, for these plots, we have used a value of m that is as close as possible to that in [8], which in itself appeared to be arbitrary; in fact, it turns out that, for this set of parameters, any value smaller than this does not affect the shape of the oscillation-mark profile.

**Figure 10.**
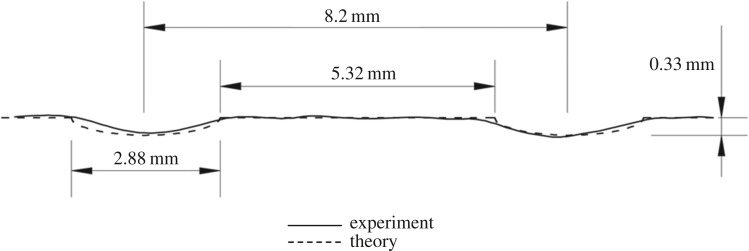
Comparison of the theoretically calculated oscillation-mark profiles and those measured experimentally by Saleem [13].

Finally, figure 10 compares the experimental and theoretical results. It shows the profile of the outer surface of the casting for two adjacent oscillation marks; the experimental result was obtained in the way explained at the end of §2. As seen already in figure 4, the experimentally obtained profiles are never exactly identical, although the average value for the pitch agrees well with the theoretical value of 2*πV*_cast_/*ω*. However, what is noteworthy here is that there is good agreement for the maximum depth of the oscillation mark and, as a consequence, for the profile itself; hence, there is good reason to believe that equation (7.2) captures the dependence of the oscillation-mark depth on the various process parameters.

## 8. Conclusions

In this paper, we have revisited an earlier model for oscillation-mark formation in the continuous casting of steel [8]. In contrast with that model, where reductions were made in an *ad hoc* fashion, we began with a non-dimensionalization of the governing equations, to determine which reductions can be justified on an order-of-magnitude basis, and which are made for the sake of convenience to enable tractability. Subsequently, reductions based on the latter were implemented to obtain a model that, somewhat surprisingly, could be solved quasi-analytically, yet which still gave qualitative agreement with the results of the original model; moreover, good quantitative agreement was obtained with experiments carried out more recently which meticulously measured the oscillation-mark depth from actual cast samples. Furthermore, our reduced model gives insight that was not available from the earlier model, the results of which had to be computed numerically; in particular, we are able to find an analytical expression for the depth of the oscillation mark, which has not been determined previously. The model is particularly attractive in view of the fact that the only other theoretical way to determine oscillation-mark profiles is via time-consuming computations involving computational fluid dynamics. Moreover, our method is valid for both sinusoidal and non-sinusoidal mould oscillations, in the sense that the expressions that we obtained for *H*_0_ in equation (6.54) and in the equation after (6.55) depend only on V; thus, these expressions hold regardless of whether a sinusoidal function, as in equation (3.2), is used for *V* , or non-sinusoidal profiles, as developed in [21–23]. A further point of note is that although our model has focused on the no-superheat limiting case, this turns out to be a very worthwhile assumption to make, for the following reasons:

— Even if we were to take a superheat as large as 50 K, for example, this would introduce a dimensionless parameter 50/(*T*_m,s_−*T*_w_)=50/(1773−300)≈0.03; this is arguably small enough to be neglected, bringing us to the no superheat case.
— It allows us to make substantial analytical headway, and essentially reduces the problem to one for the flux layer and the solid steel, which is desirable so as to avoid having to model turbulent flow in the melt.
— The fact that we have obtained such good agreement in figure 10 indicates that the assumption is not unreasonable.

In its current form, the model is very easy to use. As input data, one would need the operating parameters and the properties of the flux powder and the steel grade being cast; as regards these properties, this has recently been facilitated by the publication of two articles which document them [17,24]. Thereafter, we can note that all of the results shown here are ultimately in terms of closed-form analytical expressions. However, whether the model will give the same result as an experimental measurement is another matter. In fact, Saleem [13] also considered other steel grades where the experimental measurements did not agree with our theory; in those cases, the marks appeared to be of overflow type, and to consider this eventuality, it would be necessary to combine aspects of the models in [25,26], which take into account the meniscus, with the model presented here. It should also be noted that, whereas Hill *et al.* [8] did not consider the distinction between overflow-type and fold-type marks, which characterizes the discussion on oscillation marks in the metallurgical literature [2,14], it has become evident in the course of this study that the model in its current form addresses only fold-type marks.

The model presented here provides the framework for numerous extensions, almost all of which will most likely require numerical work. One of the simpler extensions would be the inclusion of temperature-dependent slag viscosity; data reported elsewhere indicate that this can change by around one order of magnitude in the temperature range of interest in continuous casting. Also of interest is to see how the model behaves when the convection terms that were neglected in the heat equations for the solid and molten flux are included, as the analysis indicates they ought to be; these were omitted in [8] also. A further issue is whether the expression used for the flux of the slag in the so-called upper zone—given in dimensionless form by equation (6.48)—can be improved upon; it became evident that this led to periodic closing of the slag channel, which may not be entirely realistic. This meant that one of the terms in the model—the pressure gradient between the top and bottom of the mould—was effectively never used; on the other hand, if the channel does remain open, the model behaviour must surely become much more complex, with potential feedback from the bottom of the mould to the top. Indeed, it is these factors that may account for the fact that oscillation marks are not, in practice, identical and the observation that what is often observed during continuous casting is akin to chaotic behaviour [6]; however, whereas this has sometimes been attributed to the turbulent flow emanating from flow inlet, there seems the possibility that chaotic behaviour could arise from the model equations presented here, even though the inlet has not been explicitly considered.

A final and compelling reason for pursuing the approach presented here is because it also provides a simple, yet detailed enough, framework for considering defects that are associated with oscillation marks—in particular, macrosegregation and cracks. The modelling of these would require, respectively, the inclusion of a mushy region, rather than a distinct solid–liquid interface and a thermomechanical model [16].

## Supplementary Material

papfigs7RR.m

## Supplementary Material

legend2latex.m

## Supplementary Material

laprint.m

## Supplementary Material

plotepstex.m

## Supplementary Material

Hill5.txt

## Data accessibility

The Matlab program used to generate the data for this study has been uploaded as electronic supplementary material.
